# Induction of 2n pollen with colchicine during microsporogenesis in *Phalaenopsis*

**DOI:** 10.1270/jsbbs.21100

**Published:** 2022-08-26

**Authors:** Ting Wu, Xin Zhao, Shuhua Yang, Jiahui Yang, Jun Zhu, Yaping Kou, Xiaonan Yu, Hong Ge, Ruidong Jia

**Affiliations:** 1 Institute of Vegetables and Flowers, Chinese Academy of Agricultural Sciences, Beijing 100081, China; 2 Beijing Forestry University, Beijing 100081, China

**Keywords:** meiosis stage, flower bud growth, 2n pollen, colchicine

## Abstract

The induction of 2n pollen is an important technique for breeding polyploid plants. Here, we observed meiosis in the pollen mother cells (PMCs) of six *Phalaenopsis* cultivars and attempted to induce 2n pollen. The meiotic stage was related to flower bud length. During meiosis, *Phalaenopsis* cultivars with flower widths of approximately 20–40 mm and 50–60 mm had bud lengths of approximately 3–8 mm and 5–13 mm, respectively. The duration of meiosis ranged from 4.2 to 14 d. This was the first study to characterize meiosis of the PMCs of *Phalaenopsis*. The natural generation frequency of 2n pollen varied from 0.68% to 1.78%. Meiotic stage and colchicine concentration significantly affected the induction of 2n pollen. The most effective treatment for obtaining 2n pollen was 0.05% colchicine in the leptotene to zygotene stage for 3 d, which achieved a 2n pollen frequency of 10.04%.

## Introduction

Members of the genus *Phalaenopsis* are some of the most popular potted plants in the horticultural market (Minh *et al.* 2016) because of their colorful and long-lasting flowers (Bolaños-Villegas *et al.* 2008). Orchid breeders have successfully created thousands of *Phalaenopsis* cultivars (Hsu *et al.* 2010). Chromosome doubling plays a critical role in perfecting *Phalaenopsis* horticultural traits, such as flower color and size, plant height, and stress resistance (Wongprichachan *et al.* 2013). Polyploid orchids usually have stronger stems, thicker leaves, and larger and brighter flowers and show stronger adaptability to stressful environmental conditions (Baduel *et al.* 2018, Zeng *et al.* 2020). Most wild *Phalaenopsis* species are diploid, and the number of chromosomes is 2n = 2x = 38 (Kao *et al.* 2001, Shindo and Kamemoto 1963). However, many popular commercial cultivars on the market are polyploid plants. Approximately 82% of these cultivars are tetraploid, and the rest are either triploid or aneuploid (Di *et al.* 2013, He *et al.* 2013, Zhuang *et al.* 2007). Many cultivars are produced via interspecific hybridization and intergeneric hybridization (Bolaños-Villegas *et al.* 2017, Chuang *et al.* 2013, 2014, Yuan *et al.* 2018). Differences in the frequency of diploidy and the size of chromosomes in *Phalaenopsis* species make transferring desirable genes of wild species to commercial hybrids a major challenge (Chen *et al.* 2010). Most of the progeny produced by crosses between species differing in chromosome size are sterile (Chuang *et al.* 2013, 2014). Chromosome doubling is an effective method for circumventing hybrid sterility.

Polyploid plants are mainly formed through somatic chromosome doubling and 2n gametes (Eng and Ho 2019). Tissue culture technology for *Phalaenopsis* is highly developed (Cardoso *et al.* 2020, Chen *et al.* 2009, 2010, Yeung 2017); consequently, polyploid *Phalaenopsis* can be easily induced through tissue culture with anti-microtubule reagents such as colchicine (Griesbach 1981, 1985, Putri *et al.* 2019, Tavallaie and Kolahi 2017), oryzalin (Miguel and Leonhardt 2011). and nitrous oxide gas (N_2_O) (Wongprichachan *et al.* 2013). Somatic chromosome doubling can be used to obtain many polyploid plants, as well as chimeric and aneuploid plants (Eeckhaut *et al.* 2018, Van de Peer *et al.* 2017). Polyploid offspring can also be obtained by crossing untreated plants with plants treated with colchicine (Zeng *et al.* 2020). *Phalaenopsis* is known to be able to produce 2n male gametes naturally (Griesbach 1985, Zhu *et al.* 2014). Triploid and tetraploid *Phalaenopsis* hybrids can be obtained from diploid × diploid crosses; pentaploid plants can be obtained from diploid × triploid crosses; and pentaploid and hexaploid plants can be obtained from tetraploid × tetraploid crosses (Jian *et al.* 2009). The frequency of unreduced male gametes was significantly higher in triploid *Phalaenopsis* than in diploid and tetraploid *Phalaenopsis* (Zhu *et al.* 2014). However, the natural frequency of 2n gametes is low. Furthermore, 2n pollen weakly competed with haploid pollen during fertilization, which suggests that the probability of polyploid plants being produced under natural conditions is low (Hsu *et al.* 2014, Hwang *et al.* 2020, Zhou *et al.* 2020a, Younis *et al.* 2014). Thus, artificial induction is thus required to increase the production of 2n pollen.

Currently, 2n gametes are mainly induced with chemical agents, such as colchicine, which has been widely used in *Populus* (Li *et al.* 2014, Zhou *et al.* 2020a, 2020b), *Lilium* (Luo *et al.* 2016, Sato *et al.* 2010, Zhang *et al.* 2018), *Ziziphus jujuba* (Liu *et al.* 2016, Lu *et al.* 2017), and *Eucalyptus* (Yang *et al.* 2016). Another method for increasing 2n pollen is a high-temperature treatment, which has been widely used in landscape plants, such as *Populus* (Li *et al.* 2019, Tian *et al.* 2018), persimmon (Mai *et al.* 2019, Sugiura *et al.* 2000, Xu *et al.* 2008), and *Eucommia ulmoides* (Li *et al.* 2016, Song *et al.* 2015). The induction of unreduced gametes is closely related to the meiotic period of the initial treatment, the duration of the treatment, and the temperature or concentration of colchicine (Soltis *et al.* 2015, Song *et al.* 2012, 2015). Thus, the selection of an appropriate stage of meiosis for the application of the inducing agent requires careful consideration. Few studies have examined the induction of 2n gametes in *Phalaenopsis*. 2n pollen has been obtained by treating *Phalaenopsis* Sogo Yukidian ‘V3’ and *Phalaenopsis* Tai Lin Red angel ‘V31’ young flower buds with colchicine (Hsu *et al.* 2010). In addition, polyploid offspring have been obtained by crossing untreated plants with plants treated with colchicine or N_2_O (Azmi *et al.* 2016, Wongprichachan *et al.* 2013). However, few studies have characterized the meiotic stages of pollen mother cells (PMCs), let alone evaluated the most appropriate stages for applying inducing agents for 2n pollen induction.

The aim of this study was to characterize the relationship between the meiotic stages of *Phalaenopsis* PMCs and flower bud length and identify the most effective approach for inducing the formation of 2n gametes via colchicine treatment. Our findings provide key insights that could be used to aid the polyploidy breeding of *Phalaenopsis*.

## Materials and Methods

### Plant materials

**Fig. 1.**
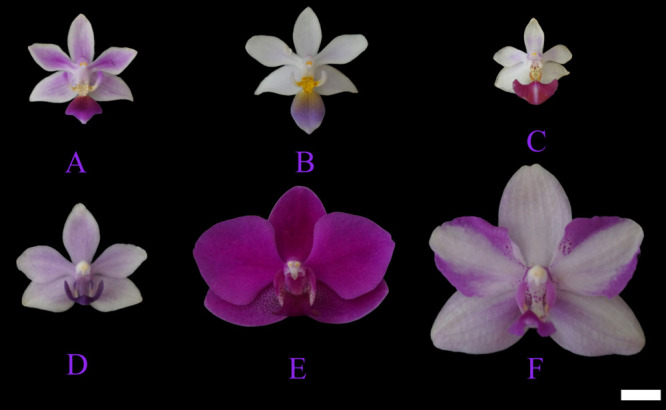
Morphology of *Phalaenopsis* cultivars. (A) *Phalaenopsis equestris*, (B) *Phalaenopsis equestris* var. *coerulea*, (C) *Phalaenopsis* Anna-Larati Soekardi, (D) *Phalaenopsis* Tzu Chiang Sapphire, (E) *Phalaenopsis* Queen Beer ‘Red Sky’, and (F) *Phalaenopsis* Purple Crystal. Scale bar = 10 mm.

*Phalaenopsis equestris*, *Phalaenopsis equestris* var. *coerulea*, *Phalaenopsis* Anna-Larati Soekardi, *Phalaenopsis* Tzu Chiang Sapphire, *Phalaenopsis* Queen Beer ‘Red Sky’, and *Phalaenopsis* Purple Crystal (Fig. 1) which are clones populations were used for meiotic process analysis. *P. equestris* was used for the induction of unreduced pollen by colchicine. All six *Phalaenopsis* plants were cultivated in a greenhouse (23–28°C) at the Institute of Vegetables and Flowers, Chinese Academy of Agricultural Sciences.

### Flower bud development and meiotic process

Photographs of three *Phalaenopsis* flower buds of each cultivar were taken at approximately 10:00 am every day from May to August 2020 to characterize changes in flower bud morphology. To prepare meiotic chromosome samples, 10–20 different sizes of flower buds were collected from 10:00 to 14:00 within a 1–2-day period three times and three times collection was completed in half a month. Morphological characteristics of *Phalaenopsis* samples were measured from the photographs. After photography, pollen samples taken from the buds were fixed in Carnoy’s solution (ethanol:acetic acid, 3:1) for 12 to 24 h at 4°C. The fixed pollen samples were then stored in 70% alcohol at 4°C. Samples were washed 2–3 times with sterile water, placed on a microscope slide, and then pressed with tweezers. A drop of lactophenol-acid fuchsin solution was added after the residues were removed. The samples were then covered with a coverslip and quickly passed through a flame 6–7 times. A pencil with an eraser was then used to lightly tap the samples on the slide to make the chromosomes evenly distributed for subsequent analysis with light microscopy (OLYMPUS DP73).

### Frequency of 2n pollen in plants grown in greenhouse and induction of 2n pollen by colchicine

Different from many other plants, *Phalaenopsis* pollen tetrads remain aggregated to each other during pollen maturation. Three flower buds with mature pollen were randomly selected for each *Phalaenopsis* cultivar, and the number of dyads, triads, and tetrads in three fields of view was counted. The percentage of 2n pollen was calculated using the following formula: 2n pollen (%) = (2Dy + Tr)/(2Dy + 3Tr + 4Te) × 100%, where Dy is the number of dyads, Tr is the number of triads, and Te is the number of tetrads.

When the PMCs were in the leptotene to zygotene, pachytene to diakinesis, metaphase I to telophase I, prophase II to telophase II, and tetrad stages, the *Phalaenopsis* flower buds were covered with cotton for 3 days and the cotton was wrapped in tin foil and had been soaked in colchicine. The colchicine concentrations used in the experiment were 0, 0.01%, 0.05%, and 0.1%. A total of 5 to 6 flower buds were used for each colchicine treatment.

### Statistical analysis

The basic data were statistically analyzed by Microsoft Excel 2017 and SPSS 2.0. Data were evaluated by analysis of variance and LSD (least significant difference) multiple comparison Significant differences were detected by Tukey’s test (≤0.05). Percentage was analyzed after inverse sine conversion before multiple comparisons.

## Results

### Relationship between meiosis stage in PMCs and the length of flower buds

**Fig. 2.**
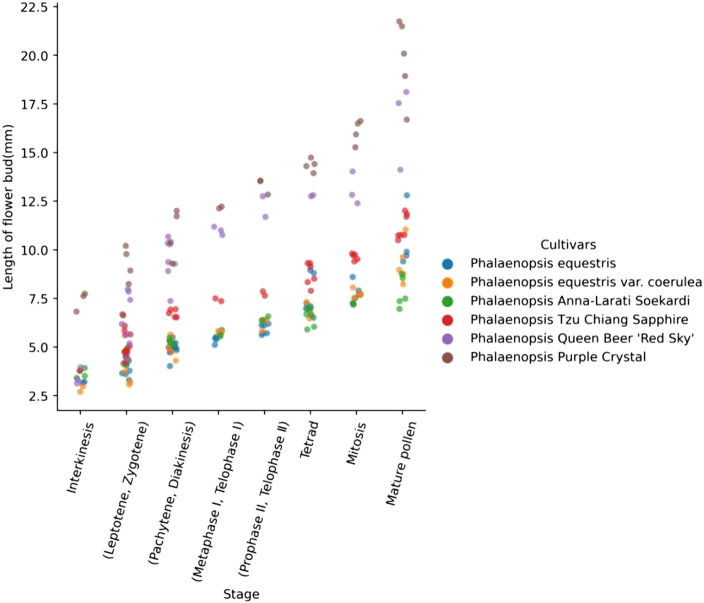
Length of the flower buds of different *Phalaenopsis* cultivars at different meiotic stages of PMCs.

**Fig. 3.**
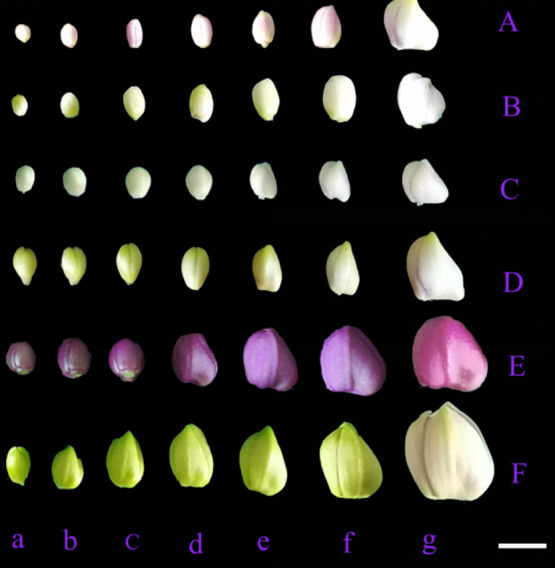
External morphology of the flower buds of *Phalaenopsis* cultivars at different developmental stages. Scale bar = 10 mm. (A) *Phalaenopsis equestris*, (B) *Phalaenopsis equestris* var. *coerulea*, (C) *Phalaenopsis* Anna-Larati Soekardi, (D) *Phalaenopsis* Tzu Chiang Sapphire, (E) *Phalaenopsis* Queen Beer ‘Red Sky’, and (F) *Phalaenopsis* Purple Crystal. (a) (Leptotene, Zygotene), (b) (Pachytene, Diakinesis), (c) (Metaphase I, Telophase I), (d) (Prophase II, Telophase II), (e) Tetrad, (f) Mitosis, and (g) Mature pollen.

Cytological observation of *Phalaenopsis* buds at different developmental stages revealed a significant correlation between the meiotic process and the length of flower buds (Fig. 2, Supplemental Table 1). The relationship between the length of *Phalaenopsis* flower buds and stages of meiosis can be clearly observed in Fig. 2. The six *Phalaenopsis* cultivars examined could be divided into two types according to the sizes of their flowers: mini *Phalaenopsis* and middle-size *Phalaenopsis*. The width of mini *Phalaenopsis* flowers (*P. equestris*, *P. equestris* var. *coerulea*, *Phalaenopsis* Anna-Larati soekard, and *Phalaenopsis* Tzu Chiang Sapphire) was approximately 20–40 mm, and the width of middle-size *Phalaenopsis* flowers (*Phalaenopsis* Queen Beer ‘Red Sky’ and *Phalaenopsis* Purple Crystal) was approximately 50–60 mm. The relationship between PMC developmental stages and flower bud external morphology in *Phalaenopsis* cultivars is shown in Fig. 3. The color of the flower buds became increasingly lighter from the leptotene stage to the mature pollen stage.

**Fig. 4.**
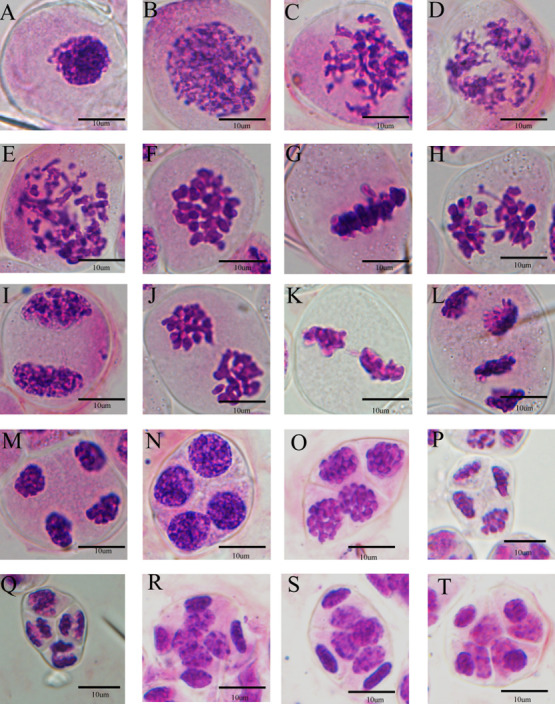
Meiotic stages of *Phalaenopsis* Anna-Larati Soekardi PMCs. (A) Pollen mother cell, (B) Leptotene, (C) Zygotene, (D) Pachytene, (E) Diplotene, (F) Diakinesis, (G) Metaphase I, (H) Anaphase I, (I) Telophase I, (J) Prophase II, (K) Metaphase II, (L) Anaphase II, (M) Telophase II, (N) Tetrad, (O) Prophase of mitosis, (P) Metaphase of mitosis, (Q) Anaphase of mitosis, (R) & (S) Telophase of mitosis, and (T) Mature pollen.

When the bud lengths of *P. equestris*, *P. equestris* var. *coerulea*, *P.* Anna-Larati soekardi, *Phalaenopsis* Tzu Chiang Sapphire, *Phalaenopsis* Queen Beer ‘Red Sky’, and *Phalaenopsis* Purple Crystal were 3.3, 3.0, 4.0, 4.2, 4.4, and 8.2 mm, respectively, the PMCs were in the leptotene stage. At this time, the chromosomes circulated, folded into thin and long lines, and aggregated into clusters (Fig. 4B). The PMCs then entered the zygotene stage, and the chromosomes shrank and thickened. The homologous chromosomes began to congregate and form the synaptonemal complex (Fig. 4C). As the length of the flower buds increased, the chromosomes formed into spirals and became shorter and thicker, and the pachytene stage began (Fig. 4D). At the diplotene stage, the chromosomes were further shortened, chromosome pairing was loosened, and synapsis could no longer be observed (Fig. 4E). When the diakinesis stage began, the spiraling of the chromosomes tightened, and the chromosomes reached their shortest length (Fig. 4F). When the flower bud lengths of *P. equestris*, *P. equestris* var. *coerulea*, *Phalaenopsis* Anna-Larati Soekardi, *Phalaenopsis* Tzu Chiang Sapphire, *Phalaenopsis* Queen Beer ‘Red Sky’, and *Phalaenopsis* Purple Crystal were 5.4, 5.6, 5.5, 7.4, 10.8, and 12.0 mm, respectively, the PMCs were in metaphase I, and the chromosomes were neatly arranged in the center of the equatorial plate (Fig. 4G). In anaphase I, homologous chromosomes began to separate and move to opposite poles via the kinetochore microtubules (Fig. 4H). When homologous chromosomes reached the opposite poles, the PMCs were in telophase I (Fig. 4I), and the chromosomes began to gradually unwind, completing the first round of meiosis. When the flower bud lengths of *P. equestris*, *P. equestris* var. *coerulea*, *Phalaenopsis* Anna-Larati Soekardi, *Phalaenopsis* Tzu Chiang Sapphire, *Phalaenopsis* Queen Beer ‘Red Sky’, and *Phalaenopsis* Purple Crystal were 5.6, 5.8, 5.9, 7.6, 11.2, and 12.9 mm, respectively, the chromatin in the nucleus was condensed and spiraled, and PMCs were in prophase II (Fig. 4J). At metaphase II, the two groups of chromosomes were arranged in the center of the equatorial plate, and most of them were parallel (Fig. 4K). When anaphase II began, the sister chromatids separated and moved via the two-stage spindle filaments (Fig. 4L). When meiosis entered telophase II, the sister chromatids gradually unscrewed, marking the end of the second round of meiosis. When the flower bud lengths of *P. equestris*, *P. equestris* var. *coerulea*, *Phalaenopsis* Anna-Larati Soekardi, *Phalaenopsis* Tzu Chiang Sapphire, *Phalaenopsis* Queen Beer ‘Red Sky’, and *Phalaenopsis* Purple Crystal were 6.2, 6.5, 6.6, 7.9, 12.8, and 13.6 mm, respectively, the PMCs were in the split stage (Fig. 4N). The flower buds then developed and underwent mitosis (Fig. 4O–4S). The flower buds finally became mature pollen, which were always in the form of tetrads (Fig. 4T).

**Fig. 5.**
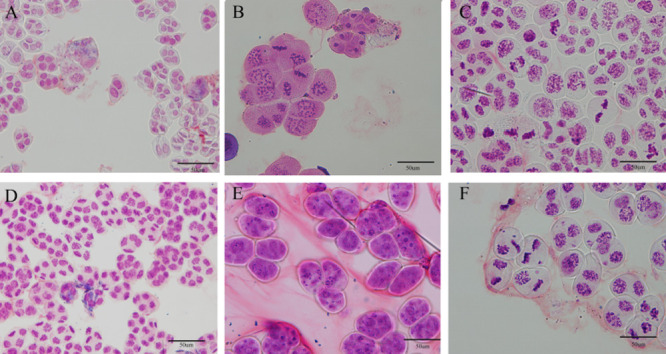
Abnormal behavior and asynchrony during the meiotic process in the PMCs of *Phalaenopsis* cultivars. (A) *Phalaenopsis equestris*, (B) *Phalaenopsis equestris* var. *coerulea*, (C) *Phalaenopsis* Anna-Larati Soekardi, (D) *Phalaenopsis* Tzu Chiang Sapphire, (E) *Phalaenopsis* Queen Beer ‘Red Sky’, and (F) *Phalaenopsis* Purple Crystal.

Although most PMCs were meiotically synchronized, meiotic asynchrony between adjacent PMCs could also observed, such as PMCs in telophase I and telophase II stages (Fig. 5B, 5C, 5E, 5F), prophase II and tetrad stages (Fig. 5A), and mitosis and mature pollen stages (Fig. 5D). In *Phalaenopsis* Queen Beer ‘Red Sky’, the synapses were in a disordered state. After meiosis was completed, no tetrads were formed, but several small nuclei were observed (Fig. 5E).

**Fig. 6.**
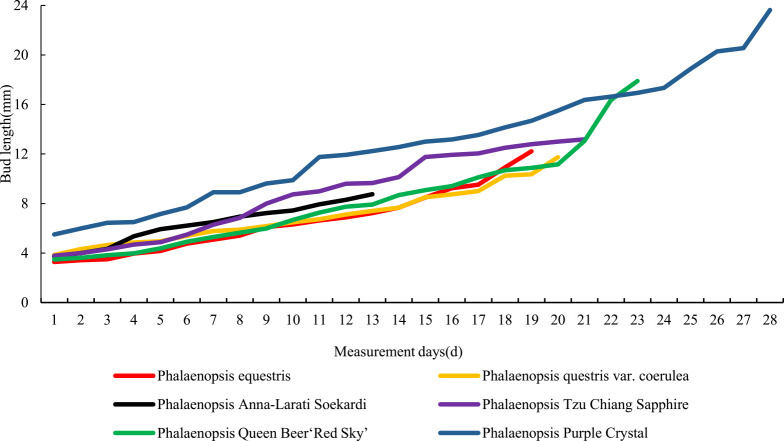
Flower bud length of different *Phalaenopsis* cultivars over time.

The growth of *Phalaenopsis* buds from the PMC stages to flowering is shown in Fig. 6. The duration of each meiosis stage could be determined based on the duration of flower bud growth and the length of flower buds at different stages (Table 1). The meiosis of *Phalaenopsis* PMCs was short in duration, and the duration of prophase I was longer compared with other meiotic stages. After meiosis was complete, the duration of the tetrad, mitosis, and mature pollen stages ranged from 4.2 to 12.7 d.

### Frequency of 2n pollen in plants grown in greenhouse and induction of 2n pollen by colchicine

**Fig. 7.**
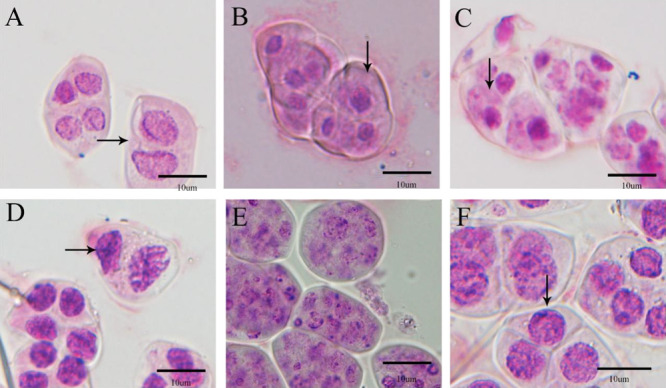
Formation of unreduced male gametes in *Phalaenopsis* cultivars. (A) *Phalaenopsis equestris*, (B) *Phalaenopsis equestris* var. *coerulea*, (C) *Phalaenopsis* Anna-Larati Soekardi, (D) *Phalaenopsis* Tzu Chiang Sapphire, (E) *Phalaenopsis* Queen Beer ‘Red Sky’, and (F) *Phalaenopsis* Purple Crystal.

With the exception of *Phalaenopsis* Queen Beer ‘Red Sky’ (Fig. 7E), dyads and triads were observed in the five other *Phalaenopsis* cultivars (Fig. 7A–D, F). The cultivars of *phalaenopsis* ploidy levels were also determined by observing the chromosome number during meiosis (Supplemental Fig. 1). The results are shown in Table 2. The natural generation frequency of 2n pollen significantly differed among cultivars and ranged from 0.68% to 1.78%. With the exception of *Phalaenopsis* Anna-Larati Soekardi, the natural generation frequency of 2n pollen did not exceed 1%.

**Fig. 8.**
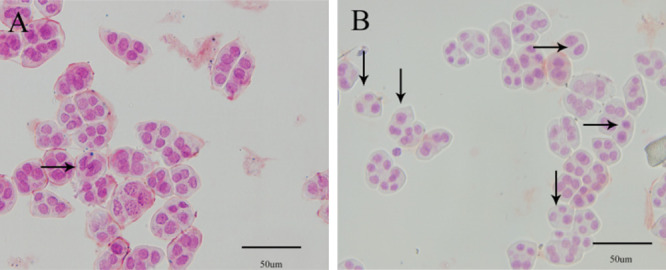
Occurrence of unreduced male gametes in *Phalaenopsis equestris* before and after colchicine treatment. (A) Control *Phalaenopsis equestris*; (B) *Phalaenopsis equestris* treated with colchicine.

Mature pollen was collected after the flower buds of *P. equestris* were treated with colchicine at different stages of meiosis. The percentages of dyads and triads and the frequency of unreduced male gametes are shown in Table 3. According to a univariate generalized linear model analysis, we found that the dominant meiotic stage, colchicine concentration, and the dominant meiotic stage × colchicine concentration interaction had highly significant effects on the frequency of colchicine-induced 2n pollen (Table 4). The percentage of dyads and triads was significantly greater when *P. equestris* was treated with colchicine than without colchicine treatment (Fig. 8). Pollen was not harvested from all treatments (Table 4), as some flower buds withered or dropped early after treatment either because the concentration of colchicine was too high or the flower buds were too small. LSD multiple-comparison tests indicated that the frequency of unreduced male gametes was significantly higher under treatment with 0.05% colchicine from the leptotene to the zygotene.

## Discussion

Unreduced gametes are common in plants and one of the main causes of polyploidy (Baduel *et al.* 2018, Eng and Ho 2019, Soltis *et al.* 2015, Song *et al.* 2012, Van de Peer *et al.* 2017). Compared with somatic polyploidization, sexual polyploidization greatly increases genetic diversity and heterosis (Brownfield and Kohler 2011, Lai *et al.* 2015, Ramanna and Jacobsen 2003, Sato *et al.* 2010, Younis *et al.* 2014, Zeng *et al.* 2020). Unreduced gametes have been reported in more than 34 plant families to date (Dewitte *et al.* 2012, Sora *et al.* 2016). For example, natural 2n gametes in citrus (Ahmed *et al.* 2020, Aleza *et al.* 2012) and *Cymbidium* (Zeng *et al.* 2020) have been used to obtain polyploid offspring.

In our study, the frequency of 2n pollen in *Phalaenopsis* cultivars grown in greenhouse were determined. In the triploid *Phalaenopsis* Queen Beer ‘Red Sky’, the synapses were in a disordered state during meiosis; consequently, no tetrads were produced, which precluded estimation of the frequency of 2n pollen. The frequency of 2n pollen in most *phalaenopsis* did not exceed 1%, which is consistent with the results of previous studies (Zhu *et al.* 2014). The frequency of 2n pollen in *Phalaenopsis* Anna-Larati soekard was 1.78%, which is greater than the value (0.59%) reported in a previous study (Zhu *et al.* 2014). According to the Search The International Orchid Register website (https://apps.rhs.org.uk/horticulturaldatabase/orchidregister/orchidregister.asp), the parents of *P.* Anna-Larati Soekardi are distantly related, which might explain the observed deviation in the frequency of 2n pollen. A previous study that measured the frequency of natural 2n pollen in nine *Cymbidium* species revealed that 2n male gamete formation frequencies varied from 0.15% to 4.30%, and the frequency of 2n pollen was higher in interspecific hybrids than in traditional cultivars (Zeng *et al.* 2020). Therefore, the frequency of 2n pollen in *Phalaenopsis* cultivars is related to plant ploidy and the genetic distance between parents.

The natural frequencies of *Phalaenopsis* 2n male gametes are low (Lai *et al.* 2015, Mercado *et al.* 1997). Therefore, artificial induction is required to increase the frequency of 2n pollen production to meet the needs of breeding programs. The induction of 2n pollen has been studied in several plants, such as *Populus* (Xi *et al.* 2011, Zhou *et al.* 2020b) and *Eucommia ulmoide* (Li *et al.* 2016, Song *et al.* 2015). The selection of an appropriate stage of meiosis for application of the inducing agent requires careful consideration (Xiang 2010). Previous studies have shown that plant meiosis is a finely regulated process in which multiple genes participate in expression at different stages and in different parts of the floral organs (Kaul and Murthy 1985). The process of meiosis of PMCs is correlated with external morphological changes in flowers (Andrada *et al.* 2019, Shao *et al.* 2019), and this has been confirmed in several plants, such as persimmon (Mai *et al.* 2019), *Populus canescens* (Zhou *et al.* 2020a), *Eucommia ulmoides* (Li *et al.* 2016), and *Dendrobium officinale* (Wang *et al.* 2017). In our study, the meiosis of microspores and flower bud growth of six *Phalaenopsis* cultivars differing in flower bud size were tracked and observed. Changes in the flower bud length were correlated with stages of meiosis in *Phalaenopsis*. Therefore, the meiotic period can be obtained by judging the bud length of *Phalaenopsis*.

The artificial induction of 2n gametes is an effective approach for chromosome doubling. Colchicine induction is one of the most commonly used methods; it has been used in various plants such as *Populus* (Zhou *et al.* 2020b), *Ziziphus jujuba* (Liu and Liu 2011, Liu *et al.* 2016, Shao *et al.* 2019), and *Dendrobium officinale* (Wang *et al.* 2017). Most studies of the 2n pollen induction have administered the inducing agent via injections. Studies of 2n pollen induction in *Cymbidium tortisepalum* var. *longibracteatum* and *Dendrobium* have shown that injection of colchicine solution into the flower buds at prophase Ⅰ resulted in the highest frequency of 2n pollen (2.65% and 6.22%, respectively) (Bin 2012, Wang *et al.* 2017). We attempted to induce 2n pollen in *Phalaenopsis* through injection of colchicine; however, the flower buds failed to grow normally, and they all withered in 2–3 d after treament. This might be explained by the fact that the buds of *Phalaenopsis* are delicate; in addition, cells and tissues might have been injured by the injections. For this reason, we chose absorbent cotton wrapping method for treatment.

Factors such as the concentration of colchicine and treatment time have a substantial effect on the efficacy of 2n pollen induction. No studies to date have examined the optimal timing of colchicine application for 2n pollen induction in *Phalaenopsis*. In *Eucalyptus*, the optimal period for colchicine application is diplotene to diakinesis and metaphase I to telophase I (Yang *et al.* 2016); by contrast, the optimal period in *Populus alba* was at the end of the leptotene to diakinesis (Li *et al.* 2014), and the optimal period for *Populus canescens* was at the pachytene stage (Zhou *et al.* 2020b). In our study, the frequency of 2n pollen in *P. equestris* was significantly increased by the application of 0.05% colchicine at the leptotene to zygotene stage, and the frequency of 2n pollen was 10.04%. This is consistent with the results of a previous study of *Populus alba* (Li *et al.* 2014). Role of 2n pollen were significant in novel *Phalaenopsis* breeding (Lee *et al.* 2020). Due to the tardy germination and poor tube growth, 2n pollen cannot compete well with n pollen (Aleza *et al.* 2012 Song *et al.* 2012). In addition to improving frequency of 2n pollen production, screening of 2n pollen is important to obtain polyploid plants, In case of sieving of pollens were effective for isolation of 2n pollen (Park *et al.* 2013, Rathore *et al.* 1991, Weisenseel and Jaffe 1976), but it is not the case in orchids due to the intrinsic nature of orchid sporogenesis, namely tetrad develops without isolation. Isolation by density gradient by Ficoll could be a solution (Baldi *et al.* 1988, Midoro-Horiuti *et al.* 1999).

.Several conclusions can be made based on observations of 2n pollen induction in *P. equestris* and meiosis of the PMCs of six *Phalaenopsis* cultivars. In mini *Phalaenopsis* (flower width: 20–40 mm), 2n pollen could be induced by covering the flower buds with 0.05% colchicine when the flower bud length was approximately 3–8 mm. In middle-sized *Phalaenopsis* (flower width: 50–60 mm), 2n pollen could be induced by covering the flower buds with 0.05% colchicine when the flower bud length was approximately 8–10 mm. Our findings provide a convenient way to obtain more 2n pollen and a new insights that could aid the polyploidy breeding of *Phalaenopsis*. The induction of 2n gametes in sterile interspecific hybrids can be used to breed completely new hybrids. The increasing number of studies examining the use of unreduced 2n gametes in different species and their genetic consequences reflect the potential value of this method for plant breeding.

## Author Contribution Statement

RDJ and HG guided the research. TW, HG and RDJ conceived and designed the study. HG had oversight and leadership responsibility for the research activity planning. RDJ had management and coordination responsibility for the research activity planning and execution. RDJ provided the research materials. TW performed the experiments. TW, JHY, and JZ collected, analyzed and deposited the data. TW and RDJ wrote the initial draft. HG critically reviewed, commented, specified and revised initial manuscript. XZ, XNY, SHY and YPK revised the manuscript. All authors have read and approved the manuscript.

## Supplementary Material

Supplemental Materials

## Figures and Tables

**Table 1.** Duration of each stage of meiosis in different *Phalaenopsis* cultivars

Cultivars	Days (d)						
	(Leptotene, Zygotene)	(Pachytene, Diakinesis)	(Metaphase I, Telophase I)	(Prophase II, Telophase II)	Tetrad	Mitosis	Mature pollen
*Phalaenopsis equestris*	5.0	2.0	0.5	2.0	2.7	3.3	4.0
*Phalaenopsis equestris* var. *coerulea*	3.0	4.0	0.5	2.0	2.0	1.3	5.0
*Phalaenopsis* Anna-Larati Soekardi	1.7	0.7	0.5	1.3	1.3	0.5	4.7
*Phalaenopsis* Tzu Chiang Sapphire	4.3	1.0	0.5	0.5	3.7	1.0	8.0
*Phalaenopsis* Queen Beer ‘Red Sky’	7.0	4.3	1.0	1.7	0.5	1.7	2.0
*Phalaenopsis* Purple Crystal	2.7	2.3	1.0	1.3	1.7	2.7	7.0

**Table 2.** Frequencies of unreduced male gametes in different *Phalaenopsis* cultivars

Cultivars	Ploidy level	Total number of pollen observed	Percentage of dyads (%)	Percentage of triad (%)	Frequency of unreduced male gametes (%)
*Phalaenopsis equestris*	Diploid	12,478	0.85 ± 0.11 b	1.91 ± 0.06 b	0.90 ± 0.04 b
*Phalaenopsis equestris* var. *coerulea*	Diploid	12,965	0.68 ± 0.11 bc	1.81 ± 0.14 b	0.80 ± 0.07 bc
*Phalaenopsis* Anna-Larati Soekardi	Diploid	12,600	1.24 ± 0.10 a	4.50 ± 0.31 a	1.78 ± 0.06 a
*Phalaenopsis* Tzu Chiang Sapphire	Diploid	11,462	0.42 ± 0.15 c	1.87 ± 0.17 b	0.68 ± 0.06 c
*Phalaenopsis* Queen Beer ‘Red Sky’	Triploid	–	–	–	–
*Phalaenopsis* Purple Crystal	Diploid	13,221	0.67 ± 0.20 bc	1.73 ± 0.74 b	0.77 ± 0.1 bc

Note: “–” in the table means data are missing; different lowercase letters in the same column indicate significant differences (P < 0.05).

**Table 3.** 2n pollen production under different colchicine treatments in *Phalaenopsis equestris*

Dominant meiotic stage of PMCs	Colchicine concentration (%)	Total number of pollen observed	Percentage of dyads (%)	Percentage of triads (%)	Frequency of unreduced male gametes (%)
Pollen mother cell	0.01	13,551	1.74 ± 0.37 f	6.40 ± 1.02 h	2.53 ± 0.27 g
	0.05	12,889	1.73 ± 0.46 f	6.77 ± 1.47 h	2.63 ± 0.44 g
	0.1	–	–	–	–
(Leptotene, Zygotene)	0.01	14,189	7.81 ± 1.33 a	11.03 ± 0.96 def	7.14 ± 0.84 bc
	0.05	11,834	8.88 ± 1.01 a	18.72 ± 1.72 a	10.04 ± 1.06 a
	0.1	–	–	–	–
(Pachytene, Diakinesis)	0.01	9,445	6.46 ± 0.86 b	11.60 ± 0.87 cde	6.53 ± 0.54 c
	0.05	11,414	6.35 ± 0.10 b	15.29 ± 1.42 b	7.53 ± 0.70 b
	0.1	12,492	2.33 ± 0.79 ef	12.54 ± 1.59 c	4.50 ± 0.78 e
(Metaphase I, Telophase I)	0.01	14,221	3.85 ± 1.15 c	9.87 ± 1.41 f	4.60 ± 0.72 e
	0.05	9,377	3.45 ± 1.28 cd	14.41 ± 1.92 b	5.63 ± 0.83 d
	0.1	9,917	1.83 ± 0.41 f	11.75 ± 1.58 cd	4.07 ± 0.31 ef
(Prophase II, Telophase II)	0.01	13,769	2.85 ± 1.09 de	8.47 ± 1.23 g	3.68 ± 0.77 f
	0.05	13,127	3.35 ± 0.95 cd	10.89 ± 1.58 def	4.61 ± 0.82 e
	0.1	9,897	2.60 ± 0.78 de	10.22 ± 1.04 ef	4.01 ± 0.57 ef

Note: “–” in the table means data are missing; different lowercase letters in the same column indicate significant differences (P < 0.05).

**Table 4.** Variance analysis of the frequency of colchicine-induced 2n pollen in *Phalaenopsis equestris* under different treatments

Source of variation	Df (Degree of freedom)	MS (Standard deviation)	F (F test statistics)	P (The value of significance)
Meiotic stage	4	0.048	193.465	0.000
Concentration	2	0.013	53.294	0.000
Meiotic stage × Concentration	6	0.002	9.053	0.000
Error	101	0.000		
