# Supplementary material for: Induction of 2n pollen with colchicine during microsporogenesis in Phalaenopsis
Source: Breed Sci. 2022 Aug 26;72(4):275–84. doi: 10.1270/jsbbs.21100 (PMC9868330; doi:10.1270/jsbbs.21100)
Supplement: Supplementary file 1 — Supplemental Materials [file 72_275_s1.pdf]

doi: 10.1270/jsbbs.21100

Supplemental Table 1. Flower width of *Phalaenopsis* cultivars and flower bud length at different meiotic stages of PMCs

Cultivars	Flower width (mm)	Flower bud length (mm)						
		(Leptotene, Zygotene)	(Pachytene, Diakinesis)	(Metaphase I, Telophase I)	(Prophase II, Telophase II)	Tetrad	Mitosis	Mature pollen
Phalaenopsis equestris	23.11± 1.00	(3.30, 4.72)	(4.72, 5.40)	(5.40, 5.62)	(5.62, 6.20)	(6.20, 7.23)	(7.23, 8.60)	(8.60, 12.28)
Phalaenopsis equestris var. *coerulea*	28.47± 1.19	(3.07, 4.37)	(4.37, 5.64)	(5.64, 5.83)	(5.83, 6.46)	(6.46, 7.53)	(7.53, 8.22)	(8.22, 11.04)
Phalaenopsis Anna-Larati Soekardi	23.12± 0.36	(4.06, 5.16)	(5.16, 5.54)	(5.54, 5.90)	(5.90, 6.57)	(6.57, 7.05)	(7.05, 7.26)	(7.26, 8.75)
Phalaenopsis Tzu Chiang Sapphire	32.47± 0.76	(4.21, 6.53)	(6.53, 7.36)	(7.36, 7.60)	(7.60, 7.89)	(7.89, 9.40)	(9.10, 9.81)	(9.81, 13.48)
Phalaenopsis Queen Beer 'Red Sky'	50.83± 0.99	(4.37, 7.96)	(7.96, 10.76)	(10.76, 11.18)	(11.18, 12.74)	(12.74, 12.82)	(12.82, 14.11)	(14.11, 18.12)
Phalaenopsis Purple Crystal	58.09± 0.73	(8.24, 10.20)	(10.20, 12.00)	(12.00, 12.84)	(12.84, 13.55)	(13.55, 14.74)	(14.74, 16.62)	(16.62, 21.50)

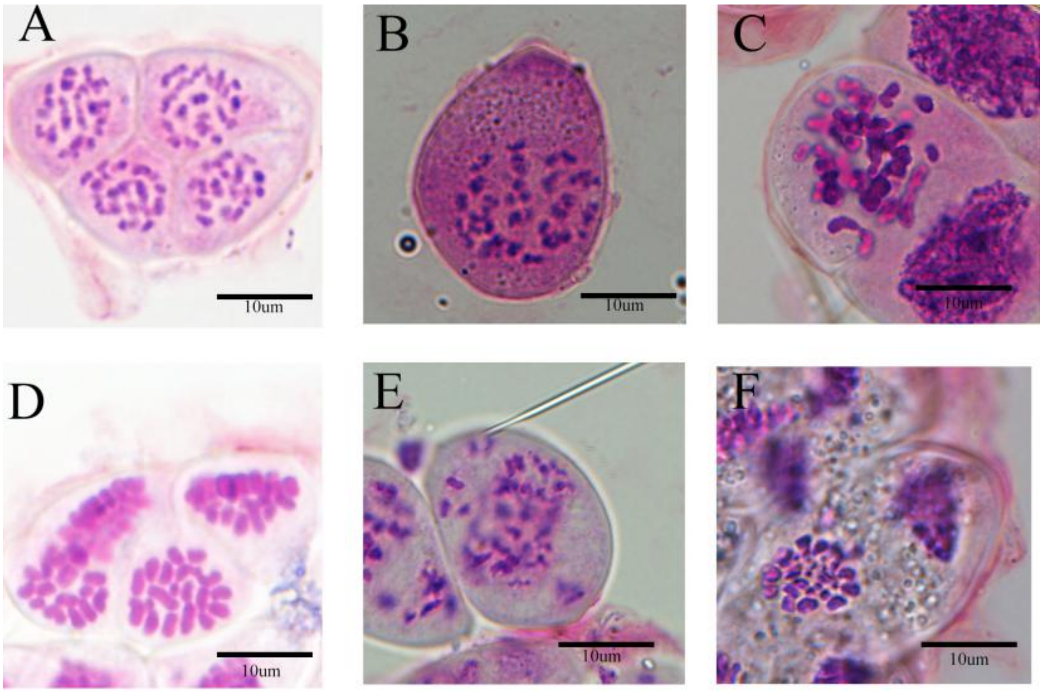

Supplemental Fig. 1. Chromosomes of *Phalaenopsis* cultivars at different meiotic stages. (A) *Phalaenopsis equestris*, (B) *Phalaenopsis equestris* var. *Coerulea*, (C) *Phalaenopsis* Anna-Larati Soekardi, (D) *Phalaenopsis* Tzu Chiang Sapphire, (E) *Phalaenopsis* Queen Beer 'Red Sky', and (F) *Phalaenopsis* Purple Crystal.
